# Secular trends in fruit intake among Danish schoolchildren, 1988 to 2006: Changing habits or methodological artefacts?

**DOI:** 10.1186/1479-5868-5-6

**Published:** 2008-01-31

**Authors:** Mette Rasmussen, Rikke Krølner, Chalida Mae Svastisalee, Pernille Due, Bjørn Evald Holstein

**Affiliations:** 1 University of Copenhagen, Institute of Public Health, Department of Social Medicine, 5 Øster Farimagsgade, P.O. Box 2099, DK-1014 Copenhagen, Denmark

## Abstract

**Background:**

Intermittent monitoring of fruit and vegetable intake at the population level is essential for the evaluation and planning of national dietary interventions. Yet, only a limited number of studies on time trends in fruit and vegetable intake among children and adolescents have been published internationally. In Denmark, national comprehensive campaigns to enhance fruit and vegetable consumption were initiated in 2001. This paper describes secular trends in fruit intake among Danish adolescents by six comparable school surveys from 1988 to 2006. The paper demonstrates and discusses the consequences of measurement changes introduced in long-term trend analyses.

**Methods:**

We used Danish data from the international Health Behaviour in School-aged Children (HBSC) study collected in 1988, 1991, 1994, 1998, 2002 and 2006. Analyses were conducted on comparable questionnaire-based data from students aged 11, 13 and 15 total (n = 23,871) from a random sample of schools. Data on fruit intake were measured by a food frequency questionnaire. Due to changes in number of response categories beween surveys, different cut-points were analysed.

**Results:**

The prevalence of students eating fruit at least once daily ranged from 78.3% among 13-year-old girls in 1988 to 17.3% among 15-year-old boys in 2002. Based on the six data collections, analyses of trends showed a significant decrease in prevalence of students eating fruit at least once daily from 1988 to 2002 (all p-values < 0.0001). In all age and gender groups, a significant increase in intake occurred between 2002 and 2006 (all p-values < 0.0065). Analyses of alternative cut-points revealed similar results.

**Conclusion:**

Fruit consumption among Danish schoolchildren decreased from 1988 to 2002 with an increase since 2002. We suggest that the increase may be attributable to a nation-wide initiative conducted in Denmark since 2001 to increase the intake of fruit and vegetables in the population. Still, the results imply that a substantial proportion of Danish schoolchildren do not meet the nationally recommended daily intake of fruit. Our analyses indicate that the observed trends are not solely caused by methodological biases related to changes in measurements.

## Background

Promoting sufficient intake levels of fruit and vegetables is important among children and adolescents for a number of reasons. First, intake of fruit and vegetables provides part of the high nutrient needs necessary for supporting rapid physiological growth and development which is characteristic for childhood and adolescence [1]. Second, food and meal habits established in childhood track into adolescence and adulthood [2,3], where they may influence health [4-9]. To evaluate national policy initiatives aimed at young people, and to assess the need for further initiatives, monitoring surveys which focus on children and adolescents are important.

In 1998, national recommendations for fruit and vegetable intake were launched. These targetted 11+ year-olds to consume fruit every day and in total to consume at least 600 g of fruit and vegetables daily. Children between the ages of 4 and 10 were to consume at least 75% [10], but this was in 2002 revised to 300–500 g per day [11]. Since the beginning of the 1990s, several nation-wide campaigns have been implemented to improve the diet of the Danish population. The most comprehensive and visible initiative focusing on fruit and vegetables is the ongoing nationwide 6 A Day campaign which is a public-private partnership with representatives from government agencies, non-governmental health organizations, and the fruit and vegetable industry. The 6 A Day campaign was launched in May 2001 following the launch of the first national recommendations for fruit and vegetable intake [12].

Only a limited number of papers on secular trends in fruit and vegetable intake among children and adolescents have been published internationally. National representative data from the US Department of Agriculture surveys showed that among US boys and girls aged 11 to 18, consumption of raw fruit (g/day measured by 24-hour recalls) declined steadily from 1965 go 1996 [13]. In a local study of 10-year-old US children in Bogalusa, Louisiana, Nicklas et al. reported an increase between 1973 and 1994 in the percentage of children who consumed fruit/fruit juices during the previous day and in mean g of fruit consumed (both based on one 24-hour recall). A significant decrease in percentage of children eating vegetables on the previous day was observed, although the mean g of vegetables consumed did not change [14]. Inchley et al. found an increase in prevalence of Scottish schoolchildren aged 11, 13 and 15 years who ate fruit and vegetables daily between 1990 and 1998, especially among girls (measured by food frequency questionnaire, FFQ) [15]. Johnson & Hackett studied the trend in intake of fruit and vegetables (eating fruit, vegetables or salad on the previous day) among 9- and 10-year-old schoolchildren living in Liverpool, England. They reported an increase in intake of fruit, vegetables and salad between 2000 and 2005 [16]. National representative data from Norway show that fruit intake among Norwegian children and adolescents aged 11, 13, 15 and 16 declined from 1993 to 2001 whereas an increase in intake was observed between 2001 and 2005 [17].

In 2003, 2004 and 2005, the Danish National Board of Health collected survey data on 11- to 15-year-olds' fruit and vegetable intake. Age-specific analyses identified a small increase in the proportion eating fruit daily among boys aged 11 (from 43% to 48%) and 15 (from 26% to 30%) and among girls aged 11 (from 57% to 64%) and 15 (from 47% to 56%) (statistical significance of change in intake are not reported). Trend analyses of intake of fruit and vegetables combined showed a significant increase among girls aged 11–15 [18]. Likewise, intake data monitored by The Danish Veterinary and Food Administration since 1995 showed that fruit and vegetable intake (g/day) among 4- to 14-year-olds' (combined age groups) increased between 1995 and 2001 [19,20]. However, it is difficult to assess long-term time trends in fruit and vegetable intake among adolescents from the above sources due to short observation periods, and varying study designs and measurements between surveys.

The assessment of trends over time requires identical measurements which are not always in accordance with the ambition to use the most recently developed and validated measurement tools. We have access to comparable data on fruit intake from repeated nation-wide representative studies of Danish schoolchildren from 1988 to 2006, which considers a long span of time before and after the launch of the 6 A Day campaign in 2001. These studies measured fruit intake by food frequency methods, but unfortunately with changes in phrasing of questions and response categories over time.

National recommendations on fruit and vegetable intake are most often provided for combined intake of fruit and vegetables. Still it is important to study trends in fruit and vegetable intake separately. Determinants of fruit intake and vegetable intake among children and adolescent may potentially be different [21] meaning that interventions may benefit from being tailored differently when aiming at fruit and vegetable intake, respectively. In both etiological and trend analyses, it is therefore relevant to differentiate between fruit intake and vegetable intake.

In the present study, we aim at describing long-term trends in the prevalence of fruit intake among Danish schoolchildren aged 11, 13 and 15 by cross-sectional data collected via six national representative and comparable questionnaire surveys between 1988 and 2006. Furthermore, we aim to demonstrate the consequences of changing measurements and to discuss to what extent observed trends reflect methodological artefacts.

## Methods

The paper reports data from the Danish study participants of the WHO cross-national Health Behaviour in School-aged Children study (HBSC) [22,23]. The overall aim of the study is to enhance the understanding of young people's health behaviours in their social settings. The study design is a series of comparable cross-sectional surveys of 11-, 13-, and 15-year-old students from random samples of schools (cluster sampling) in the participating countries with a time interval of three to four years. Data are collected by an internationally standardised self-administered questionnaire in the classroom [23].

The Danish HBSC-study comprises seven cross-sectional surveys from 1984, 1988, 1991, 1994, 1998, 2002, and 2006. All students of the relevant age groups are included from random samples of schools in Denmark. Schools which declined to participate were not replaced. All surveys were conducted by the same research team. Identical sampling approach (random sample of schools from a list of all schools in Denmark) and procedures for data collection within the classroom were applied in all surveys. For all surveys, data was collected between February and April. Every school was asked to participate by letters to the school board, the headmaster and the students' council. Data from 1984 are not available as the questionnaire applied for this survey did not include information on fruit intake. Table 1 presents descriptive data about the six surveys included in the presented analyses.

**Table 1:** Study characteristics of the six surveys

	1988	1991	1994	1998	2002	2006
A: Schools (n)	25	23	50	64	79	99
B: Participating schools (n)	18	19	45	55	68	80
School level response rate: A/B	72.0%	82.6%	90.0%	85.9 %	86.1 %	80.8%
C: Students enrolled in participating school classes (n)	1,785	2,061	4,519	5,918	5,599	7,056
D: Students present at the day of data collection (n)	1,696	1,877	4,094	5,268	4,981	6,346
E: Students included in data file (n)	1,667	1,860	4,046	5,205	4,824	6,269
Student response rate 1: E/C	93.4%	90.2%	89.5%	88.0 %	86.2 %	88.8%
Student response rate 2: E/D	98.3%	99.1%	98.8%	98.8 %	96.8 %	98.8%
Boys (n)	813	932	2,016	2,581	2,348	3,082
Girls (n)	854	928	2,030	2,624	2,476	3,187
11-year-old students (n)	526	588	1,274	1,768	1,752	2,362
13-year-old students (n)	574	643	1,414	1,859	1,619	2,222
15-year-old students (n)	567	629	1,358	1,578	1,453	1,685

### Measurements

Fruit intake was measured by one item on frequency of intake. This item was part of a FFQ with several items. The HBSC questionnaire has been used in a series of data collections in many countries and is subject to on-going validation studies. A validation study of the HBSC FFQ showed high test-retest reliability and acceptable validity when compared with data from a 24 hour food behaviour checklist and a 7 day food diary [24]. The literature shows varying results regarding the validity of self-reported dietary assessment methods among adolescents [25,26]. However, the use of FFQ to measure fruit and vegetable intake in epidemiological studies is increasingly being recognised as a valid instrument for ranking adolescents according to their usual intake [24,27]. Data generated by FFQs may thereby be less suitable for estimating prevalence levels but applicable for trend analyses and aetiological studies.

The measurements of fruit intake in the six surveys are pair-wise similar or approximately similar (Table 2). The measurements in the 1988 and 1991 surveys had identical item formulation and identical number of response categories with a deviation in the fourth and last response category. The same item formulation was kept for the 1994 and 1998 surveys (with a small deviation in 1998), while the number of response categories was increased to five. For the 2002 and 2006 surveys, the item formulation was revised and the number of response categories was increased to seven. In addition, in 2002 and 2006 the order of response keys was reversed.

**Table 2:** Item descriptions, response categories and response distributions by gender in the six surveys

**Item description**	**Response categories**	**Boys**	**Girls**
**1988:**		N = 813	N = 854
			
How often do you drink or eat the following?	More than once a day	295 (36.3)	422 (49.4)
- Fruit	Once a day	294 (36.2)	235 (27.5)
	One or several times per week	175 (21.5)	182 (21.3)
	Rarely or never	16 (2.0)	11 (1.3)
	(Missing)	33 (4.1)	4 (0.5)

**1991:**		N = 932	N = 928
			
How often do you drink or eat the following?	More than once a day	259 (27.8)	377 (40.6)
- Fruit	Once a day	348 (37.3)	283 (30.5)
	One or several times per week	280 (30.0)	231 (24.9)
	Rarely	35 (3.8)	26 (2.8)
	(Missing)	10 (1.1)	11 (1.2)

**1994:**		N = 2,016	N = 2,030
			
How often do you drink or eat the following?	More than once a day	534 (26.5)	842 (41.5)
- Fruit	Once a day	639 (31.7)	594 (29.3)
	One or several times per week	672 (33.3)	501 (24.7)
	Rarely	137 (6.8)	70 (3.5)
	Never	12 (0.6)	5 (0.3)
	(Missing)	22 (1.1)	18 (0.9)

**1998:**		N = 2,581	N = 2,624
			
How often do you eat the following?	More than once a day	577 (22.4)	843 (32.1)
- Fruit	Once a day	849 (32.9)	861 (32.8)
	One or several times per week	858 (33.2)	770 (29.3)
	Rarely	239 (9.3)	130 (5.0)
	Never	25 (1.0)	7 (0.3)
	(Missing)	33 (1.3)	13 (0.5)

**2002:**		N = 2,348	N = 2,476
			
How many times a week do you usually eat/drink.....?	Never	77 (3.3)	35 (1.4)
- Fruit	Less than once a week	217 (9.2)	159 (6.4)
	Once a week	329 (14.0)	211 (8.5)
	2–4 days a week	774 (33.0)	664 (26.8)
	5–6 days a week	321 (13.7)	417 (16.8)
	Once a day, every day	282 (12.0)	422 (17.0)
	Every day, more than once	318 (13.5)	534 (21.6)
	(Missing)	30 (1.3)	34 (1.4)

**2006:**		N = 3,082	N = 3,187
			
How many times a week do you usually eat/drink.....?	Never	99 (3.2)	33 (1.0)
- Fruit	Less than once a week	235 (7.6)	147 (4.6)
	Once a week	327 (10.6)	226 (7.1)
	2–4 days a week	873 (28.3)	666 (20.9)
	5–6 days a week	452 (14.7)	562 (17.6)
	Once a day, every day	469 (15.2)	540 (16.9)
	Every day, more than once	608 (19.7)	993 (31.2)
	(Missing)	19 (0.6)	20 (0.6)

For the 1988, 1991, 1994 and 1998 surveys, students eating fruit at least once daily were identified by the response keys, 'More than once a day' and 'Once a day'. For the 2002 and 2006 surveys, the applied response keys were, 'Every day, more than once' and 'Once a day, every day'. To evaluate the consequences of changing measurements, we also analysed an alternative cut-point for the 2002 and 2006 survey. Here, eating fruit at least once daily also included students who reported eating fruit '5–6 days a week'.

### Statistical analysis

We report un-weighted prevalence of eating fruit at least once daily and we coded missing values as not eating fruit at least once daily. Only among boys in 1988, the proportion of missing values exceeded two percent (table 2).

We used Cochrane-Armitage tests to assess significant secular trends in prevalence of eating fruit at least once daily across survey years and across age groups within surveys. Chi-square tests were conducted to test for significant prevalence differences in pair wise comparisons between surveys, and between boys and girls within surveys.

Cluster sampling causes higher standard errors compared to a similar sized sample obtained by simple random sampling of individuals. This design effect varies among items in the Danish samples [28]. A design effect of 1.5 results in 95% confidence intervals of approximately +/- 3%. Therefore, to account for the cluster sampling of individuals in schools, a level of significance of 0.02 was applied.

## Results

Table 3 presents the prevalence of students who eat fruit at least once daily stratified by survey, age and gender. The prevalence ranges from 78.3% among 13-year-old girls in 1988 to 17.3% among 15-year-old boys in 2002. Table 3 also presents prevalence estimates based on alternative cut-points for the 2002 and 2006 surveys.

**Table 3:** Proportion (%, 98% CI) of students who eat fruit at least once daily by survey, gender and age group

	**Proportion (%) of students eating fruit at least once daily**							
**Girls**	**1988**	**1991**	**1994**^c^	**1998**^c^	**2002**^c^	**2006**^c^	**2002**^ac^	**2006**^ac^
	
	n = 854	n = 928	n = 2,030	n = 2,624	n = 2,476	n = 3,187	n = 2,476	n = 3,187
	
**Age 11**	77.5 (71.0–83.2)	75.8 (69.4–81.5)	78.3^b ^(74.3–82.0)	69.1^b ^(65.4–72.6)	42.6^b ^(38.8–46.5)	51.1^b ^(47.7–54.5)	58.0^b ^(54.1–61.8)	69.8^b ^(66.7–72.9)
**Age 13**	78.3 (72.1–83.7)	70.3 (63.9–76.2)	71.0^b ^(66.9–74.9)	66.6^b ^(62.9–70.2)	36.7^b ^(32.8–40.8)	46.3^b ^(42.8–49.8)	55.5^b ^(51.4–59.6)	62.9^b ^(59.5–66.3)
**Age 15**	75.1 (68.8–80.7)	67.9 (61.6–73.7)	63.0^b ^(58.5–67.3)	58.4^b ^(54.3–62.5)	35.8^b ^(31.8–40.0)	46.2^b ^(42.2–50.2)	52.3^b ^(48.0–56.6)	63.6^b ^(59.7–67.4)

**Boys**	**1988**	**1991**	**1994**^c^	**1998**^c^	**2002**^c^	**2006**^c^	**2002**^ac^	**2006**^ac^
	
	n = 813	n = 932	n = 2,016	n = 2,531	n = 2,348	n = 3,082	n = 2,348	n = 3,082
	
**Age 11**	71.8 (64.8–78.1)	70.0 (63.4–76.0)	67.4 (62.9–71.7)	58.3 (54.3–62.2)	33.4 (29.6–37.3)	39.3 (36.0–42.8)	49.6 (45.6–53.7)	55.0 (51.5–58.4)
**Age 13**	70.8 (64.2–76.9)	61.9 (55.4–68.0)	58.8 (54.4–63.2)	59.1 (55.3–62.9)	24.7 (21.2–28.4)	35.9 (32.6–39.4)	37.2 (33.2–41.3)	50.3 (46.8–53.9)
**Age 15**	74.8 (68.1–80.8)	63.9 (57.0–70.3)	49.3 (44.8–53.8)	47.2 (42.9–51.4)	17.3 (14.1–20.9)	27.6 (24.1–31.4)	29.2 (25.3–33.4)	41.3 (37.3–45.3)

a. Alternative cut-point for categorisation of 'eating fruit at least once daily' also including students eating fruit 5–6 days a week (every day, more than once + once a day, every day + 5–6 days a week)

b. Significant gender difference within each age group (chisquare test, p< 0.02)

c. Significant trend in prevalence by age (Cochrane-Armitage tests for trend, p < 0.02)

**Figure 1:**
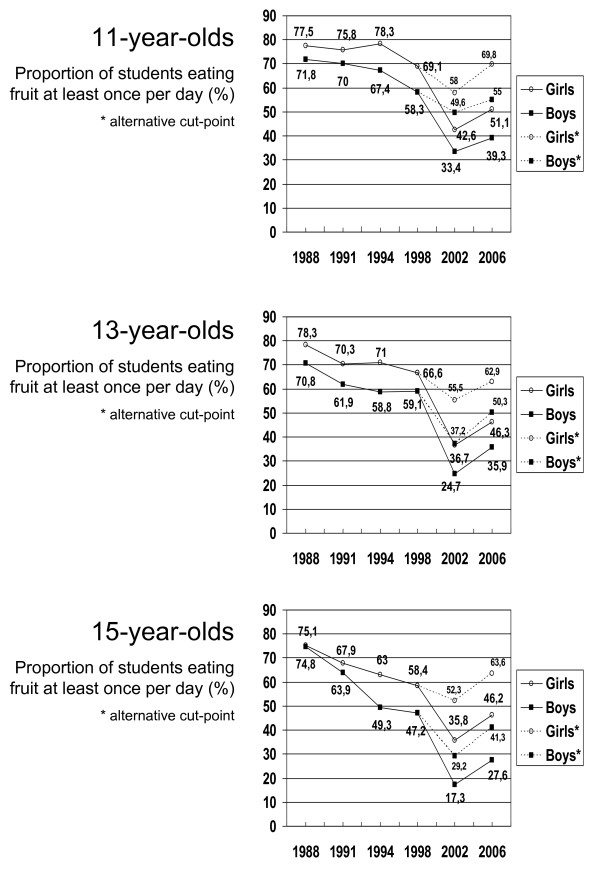


In all surveys, the prevalence of students who eat fruit at least once daily is higher among girls than boys. Only in 1988 and 1991, non-significant gender differences in prevalence exist. For all age groups, the secular trends show approximately similar patterns for boys and girls and in the majority of the surveys, the proportion of students who eat fruit at least once daily decreases with increasing age (p = 0.0039). Figure 1 presents trends in prevalence of students who eat fruit at least once daily by age and gender.

Analyses of changes in prevalence by pair-wise comparisons between surveys revealed that among 11-year-old boys and girls, the time periods from 1988 to 1991 and from 1991 to 1994, were characterised by stable fruit intake (p ≥ 0.3892), followed by a significant decrease from 1994 to 1998 (p = 0.0003). Among 13-year-old girls, a non-significant decrease in fruit intake was observed between 1988 and 1991 (p = 0.0258), followed by stable intake between 1991 and 1994 and between 1994 and 1998 (p ≥ 0.0573). Among 13-year-old boys, fruit intake decreased between 1988 and 1991 (p < 0.0186), followed by stable intake from 1991 to 1994, and from 1994 to 1998 (p ≥ 0.3522). Among 15-year-old girls, the periods from 1988 to 1991, 1991 to 1994 and 1994 to 1998 were characterised by non-significant decreases in fruit intake (p ≥ 0.0449). For 15-year-old boys, fruit intake decreased significantly from 1988 to 1991, and from 1991 to 1994 (p = 0.0050). No significant change in intake occurred between 1994 and 1998 (p = 0.4145). In all age and gender groups, fruit intake significantly decreased from 1998 to 2002 (p < 0.0001) followed by a significant increase from 2002 to 2006 (p = 0.0065). In figure 1, time periods characterised by non-significant changes in fruit intake are marked.

The 6 A Day campaign was initiated in 2001 only 10 months prior to the data collection in 2002. Therefore, separate trend analyses for the period from 1988 to 2002 were conducted. For all age and gender groups, we identified significant decreasing trends in prevalence of students eating fruit at least once daily (p-values for trend < 0.0001). Despite the significant increase in prevalence between 2002 and 2006, significant decreasing trends were also found for all age and gender groups for the complete study period 1988 to 2006 (p-values for trend < 0.0001).

Additional analyses of prevalence data based on alternative cut-points for the 2002 and 2006 survey revealed changes in the prevalence of eating fruit at least once daily, but no difference in trend patterns. For all age and gender groups, a significant decrease occurred between 1998 and 2002 (p-values ≤ 0.0146) followed by a significant increase between 2002 and 2006 (p-values ≤ 0.0101). The trends from 1988 to 2002 and for the full study period also remained significant for all age and gender groups (p-values for trend < 0.0001).

## Discussion

Our analyses indicate that since 1988, increasing proportions of Danish children and adolescents aged 11, 13 and 15 do not eat fruit daily. Thus, an alarming proportion of Danish schoolchildren do not meet the recommendations for consuming fruit every day. Nevertheless, our results do indicate a recent positive trend. For all age and gender groups, we identified a significant increase in prevalence of schoolchildren who eat fruit at least once daily between 2002 and 2006.

The Danish National Board of Health also revealed an increasing tendency in fruit intake – especially among 11- and 15-year-olds and most pronounced among boys [18]. Still, direct comparisons with our results are not feasible. Beyond the use of varying sampling approaches and FFQs, the data available from The National Board of Health were collected in 2003, 2004 and 2005 which is in the interim of the 2002 and 2006 HBSC surveys. Beyond the data presented in the present analyses, no 2006 data are available on fruit intake among Danish adolescents aged 11, 13 and 15. The next HBSC survey planned for 2009/2010 and new data from The National Board of Health will show whether the observed positive trends in adolescents' intake of fruit is an actual established upward trend.

Trend data from the Danish Veterinary and Food Administration, The Danish Institute for Food and Veterinary Research and The Danish Fitness and Nutrition Council show that the fruit and vegetable consumption of the adult population increased from 1995 to 2001 [19,20,29]. However, this increasing trend stagnated from 2001 to 2004 [29,30]. Despite differences in applied measurements, our results suggest that since the mid 1990s, adolescent and adult populations show different trend patterns in fruit intake.

The literature on gender differences in fruit and vegetable consumption among adolescents finds that girls have a higher or more frequent intake than boys, or that no gender differences exist. One body of literature finds decreasing intake of fruit and vegetables by increasing age in adolescence, while other studies find no differences in intake by age [21]. The presented findings correspond with the literature suggesting that girls eat fruit more frequently than boys and that younger students eat fruit most frequently.

Conclusions from the presented results must consider the risk of bias introduced by varying item formulations and response categories between surveys. Even small changes in wording of questionnaire items may result in different response distributions. Also changes in the wording, number and order of response categories influence the participants' responses [31,32]. Pair-wise comparisons between 1988–1991, 1994–1998, and 2002–2006 are relatively unproblematic due to identical or almost identical measurements. The most important change introduced between the surveys in 1991 and 1994 was the inclusion of a fifth response category while the wording of the question remained unchanged. It is however possible that the inclusion of an extra response category may have caused a larger spread of responses which result in a decrease in the proportion of students who report eating fruit every day. The trends from the first and second survey to the third and fourth survey may therefore partly resemble a methodological artefact.

For the 2002 survey more substantial changes were introduced in the questionnaire. Beyond the revision of the item formulation, two extra response categories were added and the order of response keys was reversed. Again, we cannot exclude that the clear decreasing trend observed between 1998 and 2002 partly reflects a methodological artefact introduced by the change of measurement for the 2002 survey. To study the consequences of these potential biases we therefore analysed an alternative cut-point for the 2002 and 2006 data. Here, we also included intake of fruit 5–6 times per week in the category 'eating fruit at least once daily'. Still, a significant decrease was observed between 1998 and 2002 for all age and gender groups suggesting that the identified decrease in prevalence of students eating fruit at least once daily from 1998 to 2002 is not solely an artefact caused by changes in the applied instrument. Still, there is a risk that the change in wording of the item (from 'how often' to 'how many times a week') reduces the comparability of data from before and after 2002 more than can be accounted for by choice of cut-point. In repeatedly conducted surveys suitable for trend analyses, we generally recommend that when unavoidable changes are introduced, old items should be kept at least for one survey. Hereby, differences due to measurement methods can be evaluated.

Another potential bias of the presented analyses is differences in availability of fruit across surveys due to seasonal differences in data collection across surveys. However, all data were collected between February and April which in Denmark is a period of the year characterised by uniform supply of fruit.

As part of the 6 A Day campaign launched in 2001, a number of initiatives aimed at increasing fruit and vegetable intake specifically among schoolchildren have been implemented between 2002 and 2006. Among others, these included voluntary subscription programmes and other efforts to increase availability of fruit and vegetables in schools. Further, games, posters and other reading materials were introduced to increase schoolchildren's awareness of the importance of eating fruit and vegetables and knowledge of dietary recommendations [12]. Between 2002 and 2006, we observed a significant increase in the prevalence of schoolchildren who eat fruit at least once a day. In contrast to the recent trend data available for the adult population [29,30], our data therefore suggest an effect of the 6 A Day campaign among schoolchildren. The measurements applied in the 2002 and 2006 surveys are completely identical and the risk of the observed increase in prevalence being an artefact due to methodological issues is therefore minimal. We cannot, however, exclude that increased awareness of the importance of fruit and vegetables introduces a social desirability bias causing an overestimation in the students' reports of intake [33]. Correspondingly, increased awareness may have caused an overestimation in reported intake due to changes in students' perception of what should be reported as fruit. The development in fruit intake among children and adolescents in Denmark resemble the situation in Norway. Here, a decrease in fruit consumption has been succeeded by a recent increase following a number of campaigns in the 1990s and the launch of official recommendations in 1996 [17].

Health promotion strategies aimed at increasing fruit and vegetable intake among children and adolescents are mainly conducted at national levels. One important tool for developing and optimising such national initiatives are international comparisons including trend data on intake from varying countries. Yet, only a limited number of papers on trends in fruit and vegetable intake among children and adolescents have been published internationally [13-17]. Although it may be difficult to compare trends shown in different studies due to varying methodological approaches, it is still important to increase the internationally published knowledge and information on trends in children and adolescents' intake of fruit and vegetables from varying national settings.

## Conclusion

Our analyses of long-term secular trends in prevalence of 11-, 13- and 15-year-old Danish schoolchildren who eat fruit at least once daily suggest that in all age and gender groups, intake decreased from 1988 to 2002. Between 2002 and 2006 intake increased. It is possible that some of the observed trends are methodological artefacts since the measurement of fruit intake changed twice over this 18-year period. The observed changes were however, not severely challenged by the change of definition of daily intake of fruit, and we believe that the observed changes reflect changing habits rather than methodological artefacts. The results of the presented analyses suggest an effect of comprehensive nationwide initiatives to increase fruit and vegetable intake among Danish children and adolescents. However, the situation in Denmark remains the same as in many other European countries [34,35], where too many schoolchildren still do not meet the national recommendation for fruit intake.

## Authors' contributions

MR, RK, BH and PD contributed to data collection and planning of research questions and analytical strategy. Data analyses were conducted by MR. MR drafted the manuscript with critical input from RK, CMS, BH, and PD. All authors have read and approved the final manuscript.
